# Pregnancy after frozen embryo transfer in mycobacterium tuberculous salpingitis: A case report and literature review

**DOI:** 10.18502/ijrm.v13i6.7288

**Published:** 2020-06-30

**Authors:** Firouzeh Ghaffari, Shokouholsadat Miralaie, Zahra Chekini, Maziar Faridi

**Affiliations:** ^1^Department of Endocrinology and Female Infertility, Reproductive Biomedicine Research Center, Royan Institute for Reproductive Biomedicine, ACECR, Tehran, Iran.; ^2^Department of Surgery, Iranmehr Hospital, Tehran, Iran.

**Keywords:** In vitro fertilization, Embryo transfer, Female genital tuberculosis, Salpingitis.

## Abstract

**Background:**

Genital tuberculosis is a common cause of infertility due to blocked and permanently damaged fallopian tubes.

**Case:**

In this case report, we describe one extremely rare case of tuberculous salpingitis in a woman who presented with infertility. She received anti-tuberculosis (TB) treatment 10 yr prior. In vitro fertilization (IVF) and intracytoplasmic sperm injection were carried out in our institute. Then, she underwent a laparoscopic salpingectomy due to bilateral hydrosalpinx and a frozen embryo was transferred, which led to pregnancy and a healthy child.

**Conclusion:**

It was concluded that IVF and frozen embryo transfer provides treatment for tubal TB with a receptive endometrium. Laparoscopic salpingectomy prior to embryo transfer plays a critical role in predicting the occurrence of a pregnancy in a patient with hydrosalpingitis attributed to TB

## 1. Introduction

Tuberculosis (TB) is an infectious disease that remains a major health problem worldwide (1). Manifestations related to the variety of TB include pulmonary and extrapulmonary forms. Genital tuberculosis (GTB) develops by hematogenous spread from extrapulmonary TB (90%), direct spread via the lymphatic system, or directly from the GI tract mesenteric nodes or peritoneum (1). Although found in different regions of the female genital tract, GTB is not usually diagnosed due to asymptomatic manifestations (1). The incidence of GTB has a wide variation, with the highest incidence in India and South Africa (19%) compared to the lowest in Australia (0.69%) (1). Latent GTB is one of the most common causes of chronic pelvic inflammatory disease (PID), menstrual abnormalities (2, 3), and infertility in women due to obstruction and permanently damaged fallopian tubes (2, 3). The genital organs commonly affected by Mycobacterium TB include the fallopian tubes (95-100%), endometrium (50-60%), ovaries (20-30%), cervix (5-15%), vulva and vagina (1-2%), and myometrium (2.5%) (1). Mucosa of the fallopian tube with or without the uterus and ovaries are involved in tubercular hydrosalpinx (2). Adverse effects on oocyte and embryonic implantation have been reported in 46% of GTB cases (3). Early diagnosis is imperative because of the effect on fertility in young, reproductive age women (2). In vitro fertilization (IVF) and embryo transfer (IVF-ET) after anti-TB treatment is the only realistic treatment in these cases, which can preserve reproductive function particularly in cases where the uterus cavity has the least damage. Despite this treatment, there is a low conception rate (19.2%) and live birth rate (7.2%) (2, 4). Most cases result in spontaneous miscarriage and ectopic pregnancy (2). The objective of the current article is to report a rare case of tuberculous salpingitis in a woman who achieved a pregnancy after a frozen embryo transfer (FET) cycle in a desperate condition. In addition, we review the literature on infertility due to GTB.

## 2. Case Report

A 30-yr-old woman with oligomenorrhea, clinical hirsutism, and a history of primary infertility for six years was referred to our institute from another province for oocyte retrieval. Laparotomy was performed 10 years prior for an ovarian cyst. The results revealed GTB and granulomatous disease with caseous necrosis. She underwent anti-TB treatment for six months.

**Figure 1:**
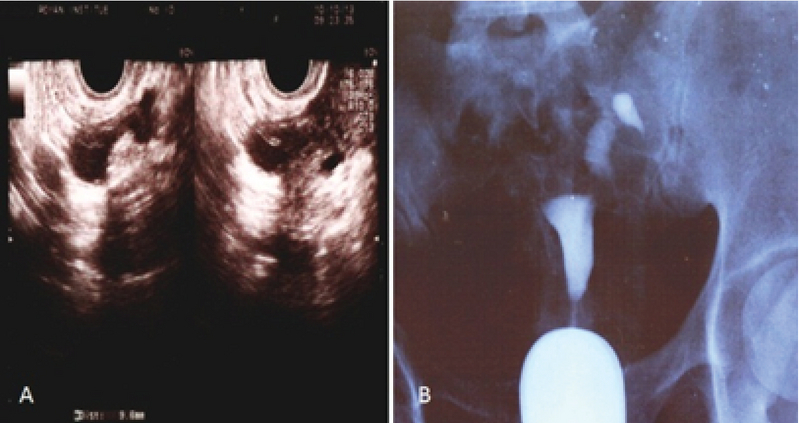
(A) Vaginal ultrasound shows a 10-mm hypoechogenic mass on the right side of the uterus. (B) Hysterosalpingography (HSG) findings in tuberculous salpingitis. HSG shows an occluded bilateral tube.

**Figure 2:**
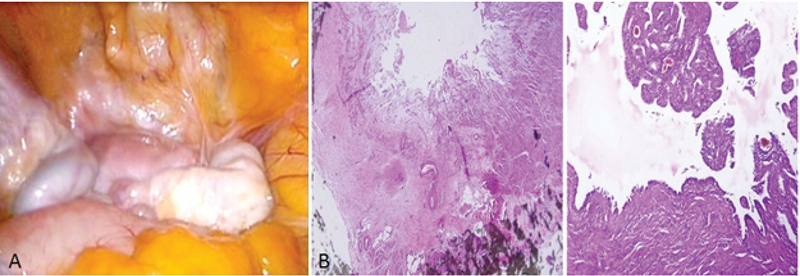
(A) Laparoscopic findings in tuberculous salpingitis. Laparoscopy showing severe dense pelvic adhesion and bilateral hydrosalpinx. (B) Histopathologic sections from fallopian tubes that shows an area of cystically dilated gland surrounded by hypertrophic smooth muscle fibers.

At the first admission in our institute, she had a negative tuberculin test (PPD) and a normal chest X-ray with no clinical evidence of TB. Hormonal evaluation on day 3 of the menstrual cycle showed a follicle-stimulating hormone (FSH) level of 6.02 mIU/ml and luteinizing hormone (LH) level of 7.2 mIU/ml. Vaginal ultrasound results revealed a 10-mm hypoechogenic structure in the right adnexa that agreed with a right hydrosalpinx (Figure 1A). Hysterosalpingography (HSG) results showed a bilateral hydrosalpinx (Figure 1B). Infertility workup for the husband indicated that he had a normal sperm analysis according to the World Health Organization criteria (5). The patient underwent ovarian stimulation according to the standard long protocol where she received 0.1 mg/day Decapeptyl (Ipsen Pharma Biotech, France) from day 20 of the pre-stimulation cycle until the day of the hCG injection. Once the down-regulation was confirmed, from day 3 of the new menstrual cycle, she received subcutaneous injections of 2 ampules/day of Gonal-F (Merck Serono, Darmstadt, Germany). Follicular growth was monitored by serial transvaginal ultrasound and repeated until the detection of at least three follicles > 18 mm in diameter. At that time, the patient received an intramuscular injection of hCG (10000 IU). After 36 hours, 21 oocytes were retrieved. Of these, 15 oocytes were fertilized and cleaved. Because of the risk for ovarian hyperstimulation syndrome, all embryos were frozen on the third day. After three months, the patient elected to undergo a laparoscopy/hysteroscopy due to hydrosalpinx (es). Laparoscopic findings included dense adhesions of the pelvic organs and a frozen pelvis. The surgeon could not perform a salpingectomy or proximal tubal occlusion so both ostium of the uterine tube were cauterized by hysteroscopy according to a previously reported procedure (6). However, direct smear and bacterial culture of endometrial tissue and evaluation of tissue by polymerase chain reaction (PCR) were negative for Mycobacterium TB. After eight months, the patient underwent FET. After GnRh agonist down-regulation, she received estradiol valerate (Aburaihan Co., Iran) as hormone replacement therapy in the following doses: 4 mg/day for the first six days, followed by 6 mg/day for another six days, until endometrial thickness reached 9 mm. When the optimal endometrial thickness was obtained, intramuscular progesterone (100 mg; Aburaihan Co, Iran) was administered and three days later, two good-quality embryos were transferred. The luteal phase was supported with estradiol and progesterone. However, she did not achieve pregnancy. One year later the patient underwent a laparoscopy. A skillful laparoscopist carried out the operation. There was a severe dense pelvic adhesion and frozen pelvis (Figure 2A). After adhesiolysis, she had a bilateral hydrosalpinx and both tubes were removed. The next day, the patient was released without any problem. Surgical specimens were collected and stained with hematoxylin and eosin and evaluated with light microscopy. We observed cystically dilated glands surrounded by hypertrophic smooth muscle fibers, which demonstrated bilateral hydrosalpingitis isthmica nodosa (Figure 2B). After one year, she underwent a second artificial FET, which was similar to the previous transfer. When the endometrial thickness reached 9.5 mm, we transferred three high-quality embryos. The luteal phase was supported by 100 mg of daily progesterone injections (Aburaihan Co., Tehran, Iran). Biochemical pregnancy was detected by a β-hCG test 2 wk after the embryo transfer and clinical pregnancy was confirmed by the presence of a gestational sac with fetal echoes at 6 wk after embryo transfer. The patient was followed and had a cesarean delivery at 38 wk of gestation. Her child was a healthy girl who weighed 3,100 g.

### Ethical consideration

The patient signed the written informed consent for reporting this case.

## 3. Discussion

GTB is a relatively rare chronic disease with unspecific symptoms of menstrual disturbance, vaginal discharge, pelvic pain, and a considerable cause of PID (4). Conversely, GTB occasionally remains undetected on clinical examination and is often diagnosed during an infertility evaluation (1).

The potential risk factors for a TB infection include a past history of TB, low socioeconomic status, HIV-seropositive persons (the highest risk among TB-infected individuals is clearly HIV co-infection, which suppresses cellular immunity), immigrants from countries with a high prevalence of TB, and Mycobacteriology laboratory personnel (7). In the current report, the patient had reported a history of TB 10 years ago, but did not have other risk factors, such as AIDS or drug addiction, that predisposed her to GTB.

HSG, laparoscopy, endometrial tissue biopsy, PCR, and histopathologic examination are useful instruments for the diagnosis of pelvic TB (3). TB has various appearances on HSG that include specific changes such as beaded tube, golf club tube, pipestem tube, cobble stone tube, leopard skin tube in addition to nonspecific changes of hydrosalpinx, tubal occlusion, hydroconvoluted tube, and tubal fixity. The effects of GTB on the endometrium may be seen as specific features and include a T-shaped uterine appearance, pseudounicornuate uterus, and collar stud abscess (3, 6). However, tubal obstruction is a common HSG finding in the majority of GTB cases (1). The HSG finding in this case was bilateral hydrosalpinx that was confirmed by laparoscopy. However, bacterial culture of the endometrial tissue and PCR were negative. In this case, the first attempt laparoscopy was not successful due to severe destruction of the tubes and pelvic adhesions. In the second laparoscopy, performed by a more experienced laparoscopist, a salpingectomy was performed with difficulty but had a favorable outcome.

The most common causes of PID are *Chlamydia trachomatis*, *Neisseria gonorrhea,* and anaerobic organisms. However, GTB may cause PID and distal tubal occlusion from salpingitis or peritubal adhesions, and subsequently hydrosalpingus (1, 3, 4). TB salpingitis can be either unilateral or bilateral, and manifested by thickened and enlarged tubal walls that turn into fibrosis and scar tissue (3). Hydrosalpinx fluid potentially has embryotoxic components and growth-inhibiting factors. The hydrosalpinx fluid may also decrease endometrial receptivity and embryo implantation. The possibility of the mechanical effect of fluid and wash-out of embryos through leakage of fluid through the endometrial cavity should be considered (8). Salpingectomy can lead to improved clinical pregnancy rate and live birth rates in patients whose infertility is the result of tuberculous salpingitis, in particular those exposed to toxic hydrosalpingeal fluid (2, 6). Dense pelvic adhesions due to salpingitis disturbances in ovarian function (2), and a higher level of FSH and LH, lower mean ovarian volume, and number of antral follicles demonstrate poor ovarian reserve in GTB (9). Possible explanations for the poor results could be endometrial insufficiency, poor response to gonadotropins, and low-quality oocytes and embryos (2, 9).

IVF procedures after anti-TB treatment have been recommended by several researchers and particularly for patients with damaged tubes and undamaged endometria (3). The pregnancy rate in such GTB cases was similar to women with infertility due to tubal factor; however, the pregnancy rate in unilateral hydrosalpinx was higher compared to bilateral hydrosalpinx (3).

Caliskan and co-workers describe GTB cases who underwent IVF cycles after antitubercular therapy and salpingectomy resulted in 15.5% clinical pregnancy per ET and 9% live birth. However, seven (33.3%) of them had hydrosalpinx and only two (9.5%) of which had severe pelvic adhesions (8). Salpingectomy before IVF cycles were reported in the underlying indication of hydrosalpinx such as ectopic pregnancy, pelvic adhesions, pyosalpinx, hematosalpinx, and hydrosalpinx (10).

Since salpingectomy has a potential risk in the frozen pelvis due to severe salpingitis, an alternative approach is cauterized ostium. In the current case, this method resulted in failed implantation. We suggested the only definitive treatment for this patient was a salpingectomy, which leads to success in implantation and lives birth.

## 4. Conclusion

Tuberculous salpingitis is a tubal cause for infertility, especially in high prevalence countries. IVF-ET provides a treatment for tubal TB with receptive endometrium. Laparoscopic salpingectomy prior to embryo transfer plays a critical role in predicting the occurrence of a pregnancy in a patient with hydrosalpingitis especially attributed to TB. GTB is a granulomatous disease where inflammation distorts the pelvic tissues, causing severe adhesions and fibrosis. An experienced laparoscopist should perform the surgery in order to prevent trauma to the pelvis and abdomen.

##  Conflict of Interest

The authors declare that they have no conflict of interest.

## References

[1] Gatongi David K, Gitau Godfrey, Kay Vanessa, Ngwenya Solwayo, Lafong Cyril, Hasan Adnan (2005). Female genital tuberculosis. The Obstetrician & Gynaecologist.

[2] S Shahzad

[3] Sharma Jai B. (2015). Current Diagnosis and Management of Female Genital Tuberculosis. The Journal of Obstetrics and Gynecology of India.

[4] N Bapna, M Swarankar, N. Kotia

[5] Wh Organization

[6] Darwish Atef M., El Saman Ali M. (2007). Is there a role for hysteroscopic tubal occlusion of functionless hydrosalpinges prior to IVF/ICSI in modern practice?. Acta Obstetricia et Gynecologica Scandinavica.

[7] Masters Barry R. (2012). Harrisons’s Principles of Internal Medicine, 18th Edition, two volumes and DVD. Eds: Dan L. Longo, Anthony S. Fauci, Dennis L. Kasper, Stephen L. Hauser, J. Larry Jameson and Joseph Loscalzo, ISBN-13: 9780071748896 McGraw Hill. Graefe's Archive for Clinical and Experimental Ophthalmology.

[8] Caliskan Eray, Cakiroglu Yigit, Sofuoglu Kenan, Doger Emek, Akar Munire E., Ozkan Sabiha O. (2014). Effects of salpingectomy and antituberculosis treatments on fertility results in patients with genital tuberculosis. Journal of Obstetrics and Gynaecology Research.

[9] Malhotra Neena, Sharma Vaishali, Bahadur Anupama, Sharma Jai B., Roy Kalol K., Kumar Sunesh (2012). The effect of tuberculosis on ovarian reserve among women undergoing IVF in India. International Journal of Gynecology & Obstetrics.

[10] Pereira Nigel, Pryor Katherine P., Voskuilen-Gonzalez Anna, Lekovich Jovana P., Elias Rony T., Spandorfer Steven D., Rosenwaks Zev (2017). Ovarian Response and in Vitro Fertilization Outcomes After Salpingectomy: Does Salpingectomy Indication Matter?. Journal of Minimally Invasive Gynecology.

